# E2-based mRNA vaccine encapsulated in lipid nanoparticles protects pigs against classical swine fever virus

**DOI:** 10.1128/jvi.00978-25

**Published:** 2025-08-21

**Authors:** Jingyi Liu, Yingju Xia, Chuanwen Tian, Ziyu Chen, Weiqiang Guo, Yingnan Liu, Jing Wen, Zhenhua Xie, Jinzhong Lin, Jiaxin Li, Hongjun Chen, Yebing Liu

**Affiliations:** 1 WOAH/National Reference Laboratory for Classical Swine Fever, China Institute of Veterinary Drug Control 620909, Beijing, China; 2 Shanghai Veterinary Research Institute, Chinese Academy of Agricultural Sciences 118161 , Shanghai, China; 3 National Key Laboratory of Veterinary Public Health Security, College of Veterinary Medicine, China Agricultural University 630101 , Beijing, China; 4 State Key Laboratory of Genetic Engineering, School of Life Sciences, Zhongshan Hospital, Fudan University 98433, Shanghai, China; University of Michigan Medical School, Ann Arbor, Michigan, USA

**Keywords:** mRNA vaccine, CSFV, E2 protein, protective immunity

## Abstract

Classical swine fever (CSF), caused by the classical swine fever virus (CSFV), remains a significant threat to the global pig industry. Recent advances in mRNA vaccines offered a promising platform for combating CSFV. In this study, we designed and evaluated three lipid nanoparticle (LNP)-encapsulated mRNA vaccine candidates encoding the ectodomain of E2 glycoprotein (E2_EX), E2_EX fused with the transmembrane (TM) region of the PEDV S protein (E2tm), and E2_EX fused with the TM region of the influenza virus HA protein (E2tm-HA). Among these, the E2tm mRNA vaccine induced the most robust antibody responses in pigs. Immunization of piglets with the E2tm mRNA vaccine showed that its immunogenicity was not impaired by maternal antibodies. Comparative analysis of pseudouridine (Ψ)-modified (^Ψ^E2tm) and unmodified (E2tm) mRNA vaccines revealed that E2tm induced significantly higher antibodies titers than ^Ψ^E2tm. All vaccinated pigs survived the CSFV challenge, with the 150 µg E2tm dose providing optimal protection, effectively suppressing viremia and preventing viral dissemination to tissues while also resulting in undetectable viral RNA in swab samples. Our findings provide a promising novel mRNA vaccine that could be used as an alternative vaccination strategy against CSFV infection.

**IMPORTANCE:**

Classical swine fever virus (CSFV) remains a significant threat to the global pig industry. While live attenuated and subunit vaccines are currently in use, there is an urgent need for more effective and safer vaccination strategies. Here, we present a novel mRNA vaccine encoding the CSFV E2 glycoprotein, which provides protective immunity against the CSFV challenge in pigs. Our findings underscore the promising efficacy of this mRNA-based vaccine platform and offer an alternative strategy for CSFV prevention and control.

## INTRODUCTION

Classical swine fever (CSF), caused by classical swine fever virus (CSFV), poses a significant threat to the global pig industry (1–3). It was first reported in America and then became endemic across Europe, South America, and Asia, causing significant economic losses (4). CSFV, belonging to the genus *Pestivirus* within the *Flaviviridae* family, is an enveloped RNA virus containing a positive-sense, single-stranded RNA genome of approximately 12.3 kb. The viral genome encodes a polyprotein, which is cleaved into four structural proteins: a core protein (C) and three envelope glycoproteins (E^rns^, E1, and E2), and eight non-structural proteins (5, 6). Among these, the E2 glycoprotein is a multifunctional glycoprotein. It plays an important role in viral entry and replication (7–9) and is also associated with viral pathogenicity in pigs (10, 11). In addition, the E2 glycoprotein is immunogenic and can induce high levels of neutralizing antibodies (12), making it an ideal target for vaccine development.

There are three major genotypes of CSFV, including 1, 2, and 3, with each genotype comprising several sub-genotypes (13, 14). In China, the predominant CSFV strains belong to sub-genotypes 1.1, 2.1, 2.2, and 2.3 (15–17). While the modified live vaccine (MLV) C-strain has been widely used with good efficacy, its effectiveness can be affected by factors such as maternal antibodies (18). A recent study demonstrated that sporadic outbreaks have occurred in C-strain vaccinated pigs (19). In addition, the MLV C-strain lacks a serological marker to differentiate infected from vaccinated animals (DIVA). Although subunit vaccines have been developed, they require higher doses to achieve protective immunity comparable to that of live-attenuated vaccines (20). Therefore, the development of novel vaccines would be highly beneficial for the prevention and control of this disease.

mRNA vaccines have shown great promise as a vaccine strategy and have been extensively explored since their success against severe acute respiratory syndrome coronavirus 2 (SARS-COV-2) (21). The use of mRNA offers several advantages. mRNA vaccines can encode various antigens, and protein production can be enhanced by engineering the untranslated regions (UTRs) of the mRNA sequences (22–24). mRNA vaccines offer potential safety advantages, as their transient expression minimizes risks of insertional mutagenesis and genomic integration (25, 26). mRNA vaccines possess self-adjuvant properties and can induce both humoral and cellular responses (27, 28). The manufacturing process for mRNA vaccines is relatively straightforward and scalable (29), allowing rapid development and adaptation for various infectious diseases. Several mRNA vaccine candidates targeting swine diseases have been explored, including those expressing the African swine fever virus (ASFV) K205R and p30 proteins (30), Nipah virus (NiV) soluble G glycoprotein (31), porcine reproductive and respiratory syndrome structural proteins (32), porcine epidemic diarrhea virus (PEDV) spike protein (33), and Japanese encephalitis virus prM and E proteins (34).

In this study, we designed three mRNA vaccines based on the CSFV E2 glycoprotein amino acid sequence. These mRNAs were successfully expressed and formulated in lipid nanoparticles (LNPs). We found that immunization with the E2tm mRNA vaccine induced the most robust antibody responses, and its immunogenicity was not affected by maternal antibodies. Additionally, we evaluated the immunogenicity and protective efficacy of both unmodified (E2tm) and pseudouridine-modified (^Ψ^E2tm) mRNA vaccines against the CFSV challenge in pigs. Notably, all vaccinated pigs survived the challenge, with the 150 µg E2tm dose demonstrating superior immunogenicity and providing optimal protection against viral infection.

## RESULTS

### Design and preparation of mRNA vaccines

**Fig 1:**
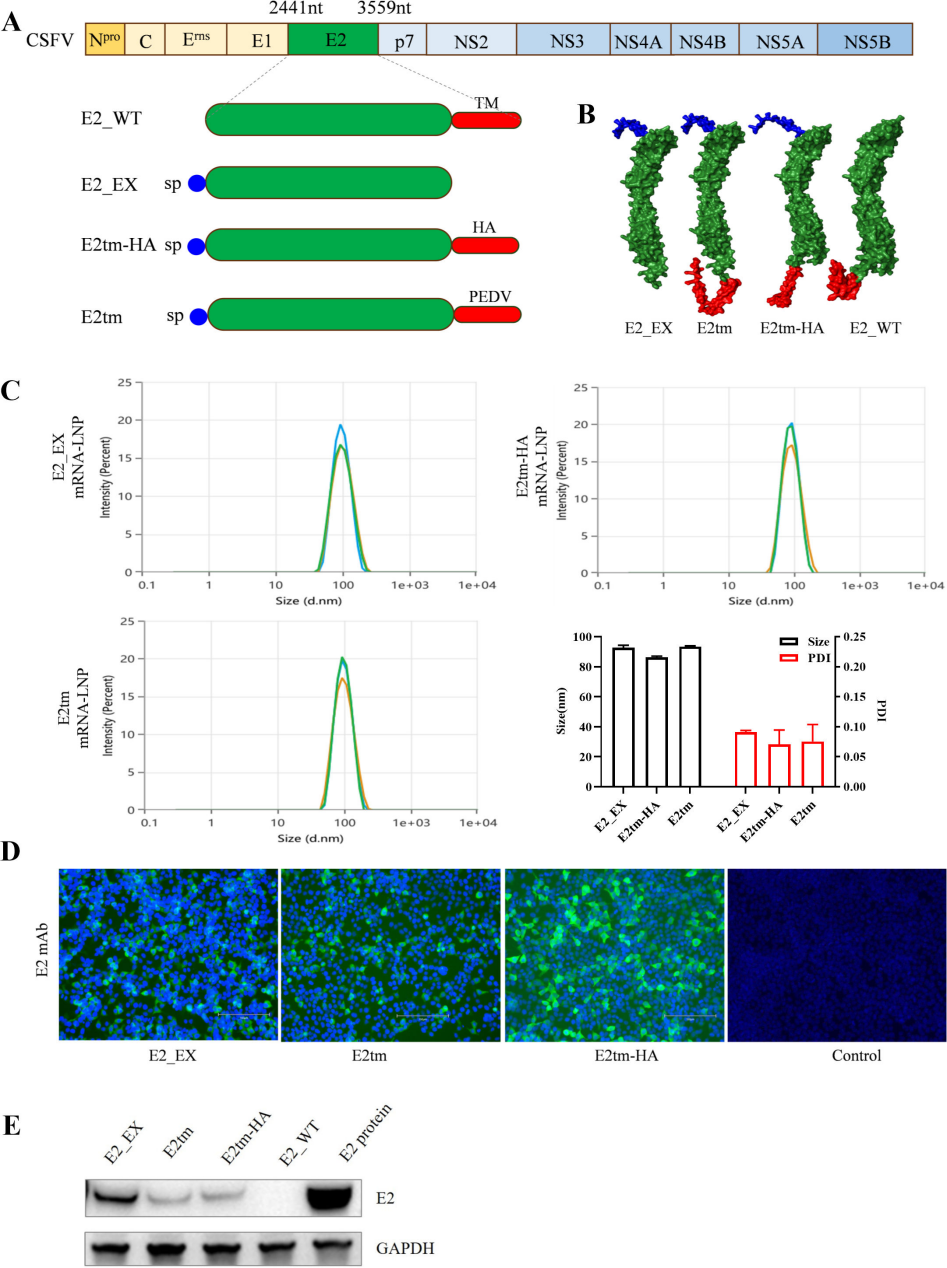
Generation of mRNAs encoding E2 glycoprotein. (**A**) Illustration of three mRNA constructs encoding the E2 glycoprotein. E2_EX contains a signal peptide (sp) and ectodomain of E2 glycoprotein. E2tm_HA and E2tm include the E2_EX and a transmembrane (TM) region of either HA protein of influenza virus or S protein of PEDV. (**B**) Structures of three mRNA encoding E2 glycoprotein. (**C**) Particle size and polymer dispersity index (PDI) graph of mRNA-LNPs. The particle sizes and PDI were measured using dynamic light scattering on a Malvern Zetasizer Nano-ZPS (Malvern). (**D and E**) Expression of mRNA-LNPs *in vitro*. HEK293T cells were incubated with mRNA-containing LNPs for 24 h and analyzed by immunofluorescence assays (IFA) (**D**) and western blotting (**E**).

Based on the E2 glycoprotein amino acid sequence, we designed three mRNA sequences encoding the E2 ectodomain (E2_EX), the E2_EX fused with the PEDV S2 protein transmembrane region (E2tm), and the E2_EX fused with the influenza virus HA transmembrane region (E2tm-HA). For all three mRNA sequences, a native E2 signal peptide was added at the N terminus (Fig. 1A). The structures of these three E2 glycoproteins are shown in Fig. 1B. Following *in vitro* transcription, the E2_EX, E2tm, and E2tm-HA mRNAs were encapsulated into LNPs. The resulting E2_EX mRNA-LNP had an average particle size of 92.75 nm and a PDI of 0.091, the E2tm mRNA-LNP had an average particle size of 93.42 nm and a PDI of 0.076, and the E2tm-HA mRNA-LNP had an average particle size of 86.2 nm and a PDI of 0.071 (Fig. 1C). The protein expression of these mRNA-LNPs was verified by detecting the E2 protein in the mRNA-transfected HEK293T cells. Immunofluorescence assay (IFA) and western blotting results confirmed that all three mRNAs were successfully expressed in HEK293T cells (Fig. 1D and E).

### Immunogenicity of the E2_EX, E2tm, and E2tm-HA mRNA vaccines in pigs

**Fig 2:**
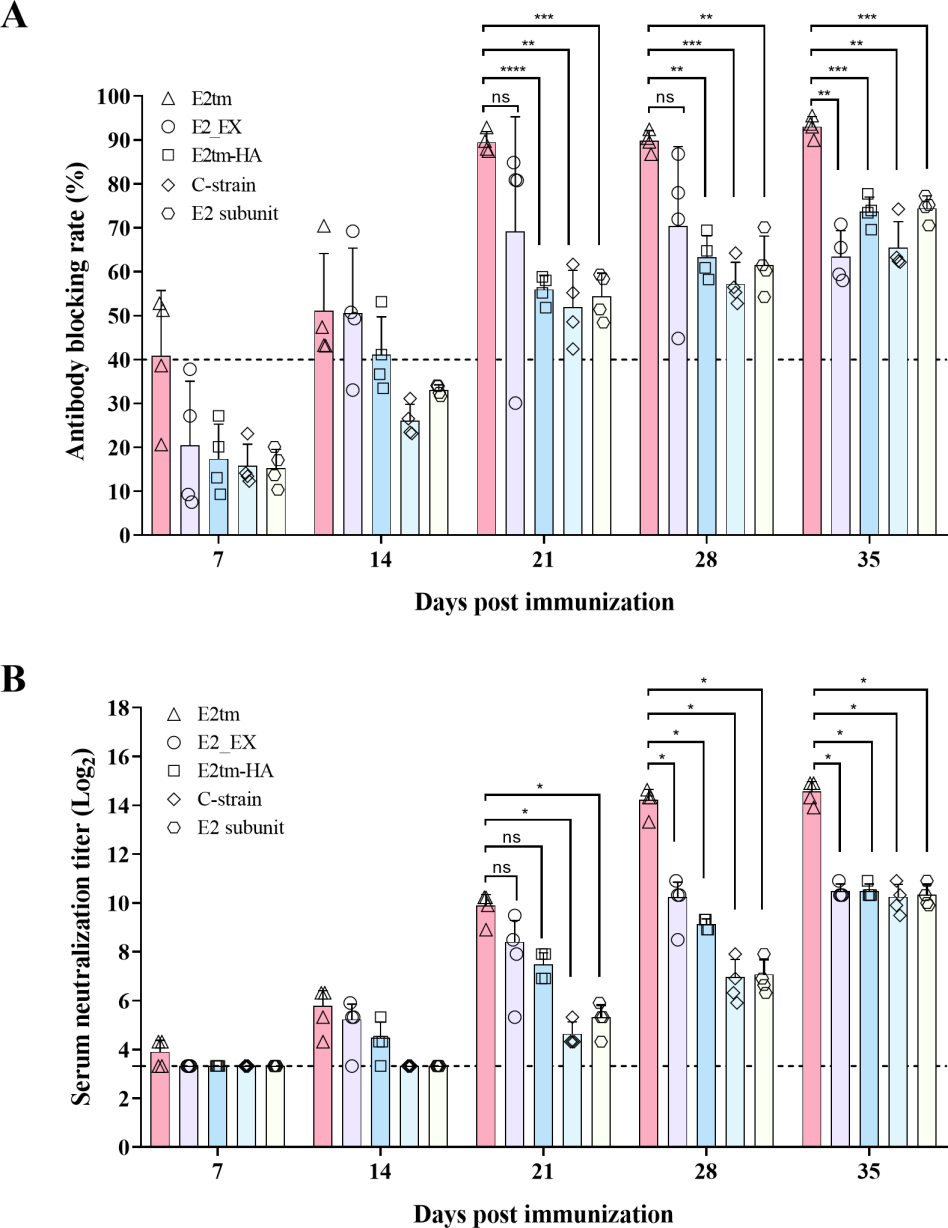
Evaluation of CSFV-specific antibodies induced by E2 mRNA vaccines in pigs. (**A**) Antibody levels in pigs immunized with mRNA vaccines. Pigs were immunized with E2tm, E2_EX, and E2tm-HA mRNA vaccines, C-strain and E2 subunit vaccines, respectively. At 7, 14, 21, 28, and 35 days post immunization (dpi), CSFV-specific antibodies in serum samples were determined by the CSFV antibody test kit. Antibody blocking rate ≥40% was considered CSFV-specific antibody positive. (**B**) The serum-neutralizing antibodies against CFSV. The neutralizing antibody titers were determined as the highest serum dilution that protected >50% of the cells from infection (ND_50_). Serum was considered neutralizing antibody-positive when the titer was ≥10 ND_50_. The dot line indicates the lower limit of detection. The data were analyzed using the two-way ANOVA and presented as the mean ± SD; ns, not significant; *P < 0.05; **P < 0.01; ***P < 0.001; ****P < 0.0001.

We compared the immunogenicity of the E2tm, E2_EX, and E2tm-HA mRNA vaccines with the attenuated C-strain vaccine or a commercial E2 subunit vaccine from Jinyu Group in pigs. The pigs were immunized with 60 µg of either E2tm, E2_EX, E2tm-HA mRNA vaccines, E2 subunit vaccine, or one dose of the attenuated C-strain vaccine. Serum samples were collected at 7, 14, 21, 28, and 35 days post-immunization (dpi) and tested for the CSFV antibodies using a blocking ELISA kit. At 7 dpi, two pigs in the E2tm group tested positive for CSFV-specific antibodies. At 14 dpi, all the pigs, in the E2tm group, three pigs in the E2_EX group, and two pigs in the E2tm-HA group tested positive for CSFV-specific antibodies. In contrast, none of the pigs with the E2 subunit vaccine or C-strain vaccine is positive (above 40%) before 14 dpi. The positivity rate continued to increase for CSFV-specific antibodies, reaching approximately 90% in the E2tm group at 35 dpi, compared to 60%–70% in the other groups (Fig. 2A). Subsequently, neutralizing antibodies (NAb) were also detected. The results showed that the NAb titers were significantly higher in the E2tm group than that in the other groups after boosting (Fig. 2B). These data suggest that the E2tm mRNA vaccine elicits a stronger immune response when compared to the E2_EX and E2tm-HA mRNA vaccines, and E2 subunit and C-strain vaccines.

### Antibody responses induced by the E2tm mRNA vaccine in piglets

**Fig 3:**
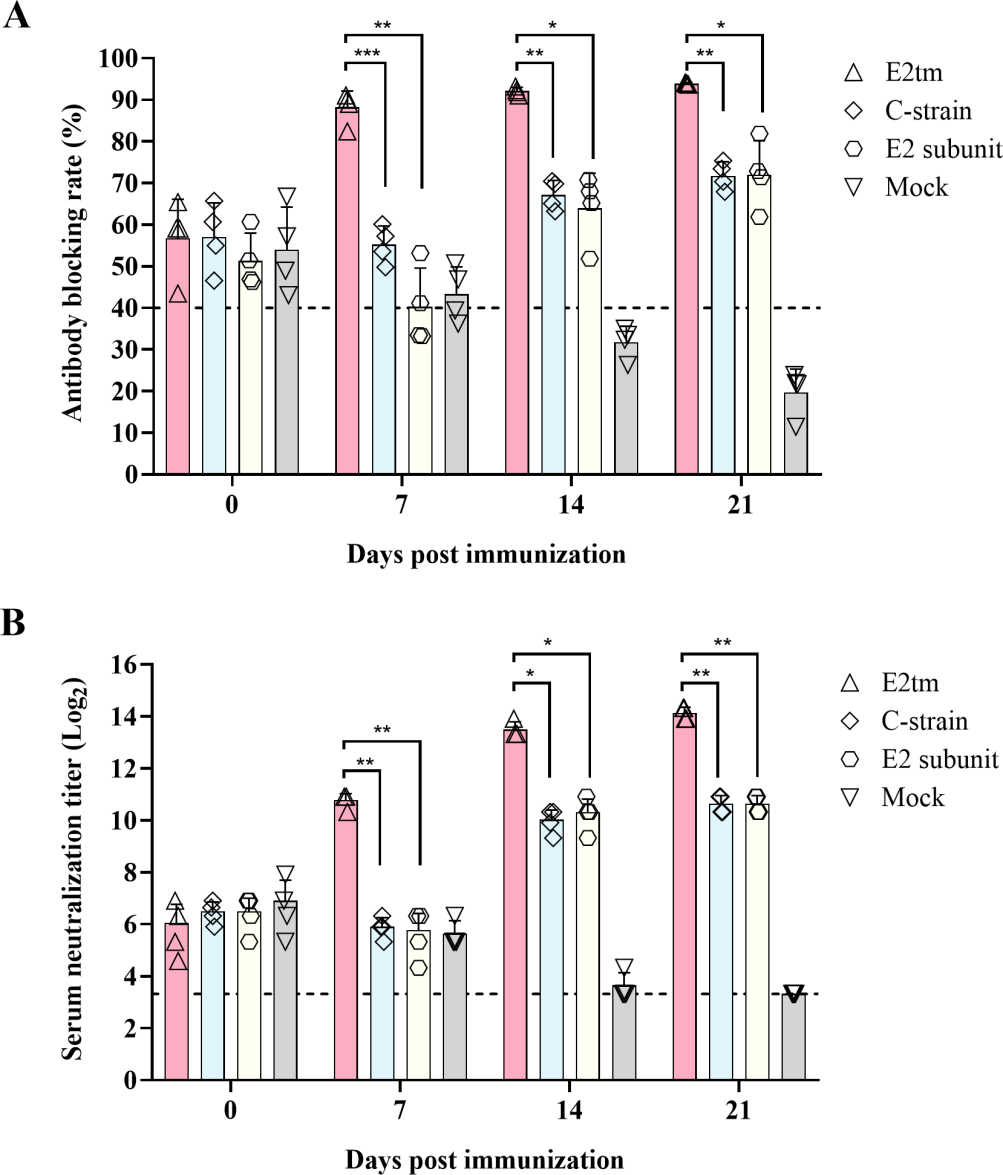
The effect of maternal antibodies on antibody levels induced by the E2tm mRNA vaccine. (**A**) Antibody levels in piglets immunized with E2tm mRNA, C-strain, or E2 subunit vaccines or mock immunized. At 0, 7, 14, and 21 dpi, CSFV-specific antibodies in serum samples were determined by the CSFV antibody test kit. Antibody blocking rate ≥40% was considered CSFV-specific antibody positive. (**B**) The serum-neutralizing antibodies against CFSV. The neutralizing antibody titers were determined as the highest serum dilution that protected >50% of the cells from infection (ND_50_). Serum was considered neutralizing antibody-positive when the titer was ≥10 ND_50_. The dot line indicates the lower limit of detection. The data were analyzed using the two-way ANOVA and presented as the mean ± SD; *P < 0.05; **P < 0.01; ***P < 0.001.

Next, we investigated whether the immunogenicity of E2tm mRNA was affected by maternal antibodies in piglets. The piglets were immunized with 60 µg of the E2tm mRNA vaccine, an E2 subunit vaccine, or C-strain vaccine. The remaining piglets were immunized with phosphate-buffered saline (PBS). Serum samples were collected for the detection of CSFV antibody levels at 0, 7, 14, and 21 dpi. As shown in Fig. 3A, all the pigs tested positive for CSFV-specific antibody prior to immunization. At 7 dpi, the positivity rate of CSFV-specific antibodies in the E2tm group increased rapidly, reaching approximately 90%, and remained high thereafter. This suggests that immunogenicity induced by the E2tm mRNA vaccine was not affected by maternal antibodies. In contrast, the positivity rate of CSFV-specific antibodies in the E2 subunit vaccine group decreased at 7 dpi before gradually increasing to approximately 70%. Evaluation of the induced NAb showed that the NAb titers were significantly higher in the E2tm group than the E2 subunit or C-strain group at 14 and 21 dpi (Fig. 3B). These data suggest that the E2tm mRNA vaccine induced a robust antibody response in piglets without interference from maternal antibodies.

### Evaluation of the effect of nucleotide modification on the E2tm mRNA vaccine

**Fig 4:**
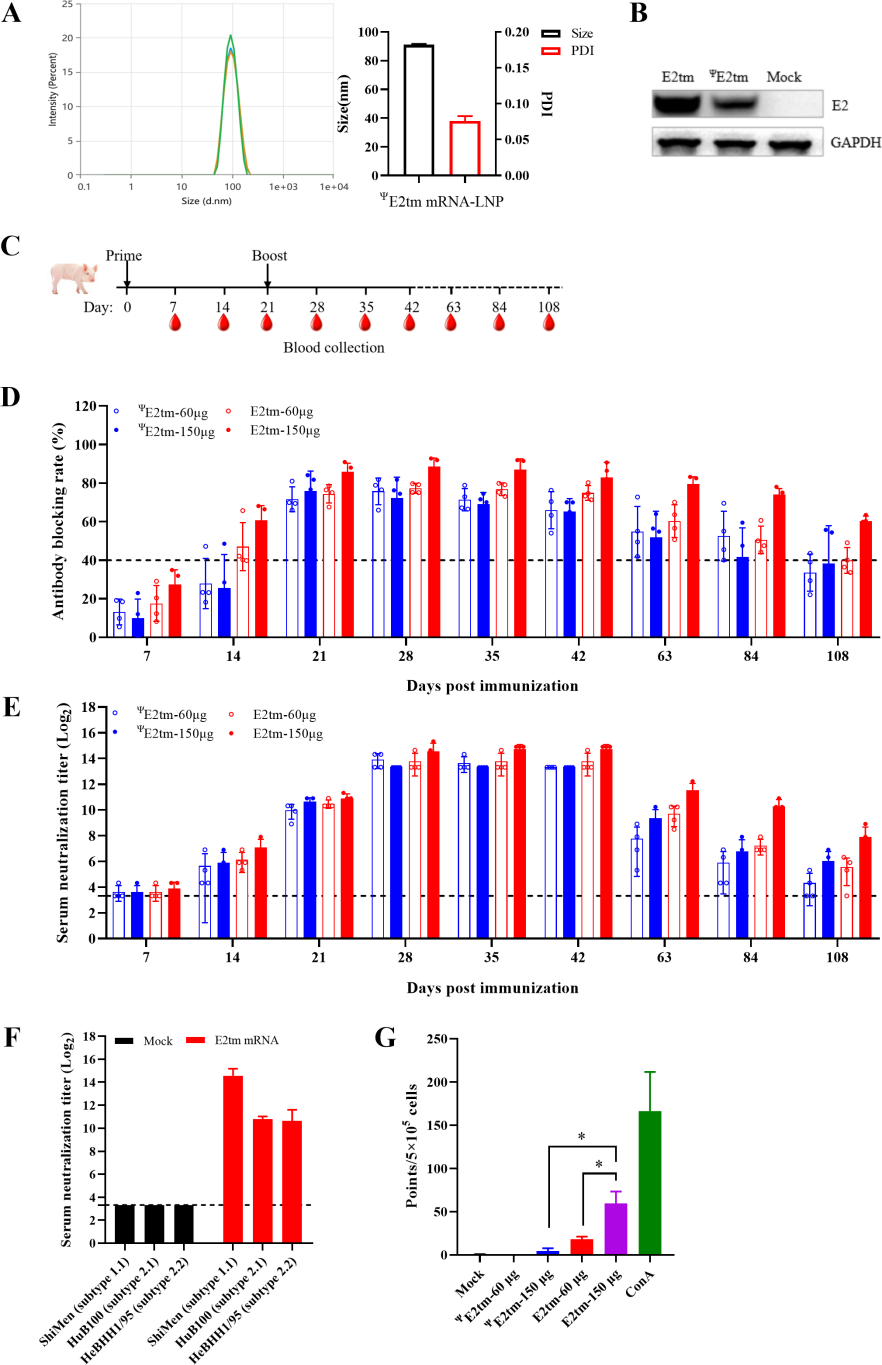
Effect of pseudouridine modification on E2tm mRNA vaccine. (**A**) Particle size and polymer dispersity index (PDI) graph of ^Ψ^E2tm mRNA-LNP. The particle sizes and PDI were measured using dynamic light scattering on a Malvern Zetasizer Nano-ZPS (Malvern). (**B**) Expression of E2tm and ^Ψ^E2tm mRNA-LNPs *in vitro*. HEK293T cells were incubated with mRNA-containing LNPs for 24 h and analyzed using western blotting. (**C**) The schematic representation of pig immunization. Pigs were immunized at days 0 and 21 with E2tm (60 or 150 µg) or ^Ψ^E2tm (60 or 150 µg) mRNA. (**D**) Antibody levels induced by E2tm and ^Ψ^E2tm mRNA in pigs. Serum samples were collected at 7, 14, 21, 28, 35, 42, 63, 84, and 108 dpi. The levels of CSFV-specific antibodies in serum samples were determined by blocking ELISA. (**E**) Serum neutralizing antibodies against CSFV Shimen strain over times. The titers were expressed as the highest serum dilution that protected more than 50% of the cells from infection. (**F**) The serum neutralizing antibodies against different CSFV strains, including Shimen strain, HuB100 strain, and HeBHH1/95 strain. (**G**) IFN-γ-secreting peripheral blood mononuclear cells (PBMCs) induced by E2tm and ^Ψ^E2tm mRNA vaccines in pigs detected by ELISpot. PBMCs were isolated from serum samples collected at 28 dpi. The dot line indicates the lower limit of detection. The data were analyzed using the one-way ANOVA and presented as the mean ± SD; *P < 0.05.

Modified nucleotides, such as pseudouridine, have been reported to enhance protein translation (35). Here, we generated a pseudouridine (Ψ) modified E2tm mRNA (designated ^Ψ^E2tm) to evaluate the effect of nucleotide modification on the E2tm mRNA vaccine. The resulting ^Ψ^E2tm mRNA-LNP showed an average particle size of 91.05 nm and a PDI of 0.076 (Fig. 4A). Protein expression of ^Ψ^E2tm mRNA-LNP was confirmed by western blotting (Fig. 4B).

To investigate the effect of the nucleotide modification on the immunogenicity of the E2tm mRNA vaccine, we immunized pigs with 60 or 150 µg E2tm mRNA and 60 or 150 µg E2tm mRNA. A booster immunization with the same dose was administrated at 21 days after initial immunization (Fig. 4C). Serum samples were collected at 7, 14, 21, 28, 35, 42, 63, 84, and 108 dpi to evaluate the levels of CSFV-specific and neutralizing antibodies. The results showed that the positivity rates of CSFV-specific antibodies in all four groups significantly increased at 21 dpi and remained high for the following 3 weeks. Although the positivity rates gradually declined in all groups, antibodies in the 150 µg E2tm group persisted at relatively high levels and remained CSFV-positive (Fig. 4D). Notably, significantly higher neutralizing antibody titers were detected after both initial and booster immunization. The neutralizing antibody titers remained high between 28 and 42 dpi and then gradually decreased but remained CSFV-positive in the 150 µg E2tm group (Fig. 4E). Neutralizing antibodies against different CSFV strains were also evaluated. The serum samples showed neutralizing activity against both the HuB100 strain (subtype 2.1) and HeBHH1/95 strain (subtype 2.2) (Fig. 4F), demonstrating that E2tm mRNA exhibited relatively broad neutralizing activities.

Next, IFN-γ ELISpot assay was conducted to compare cellular immune responses induced by these E2tm mRNA vaccines. Peripheral blood mononuclear cells (PBMCs) were isolated from pigs at 28 dpi for the ELISpot assay. The results showed that the number of PBMCs secreting IFN-γ in the 150 µg E2tm group was significantly higher than that in the 60 µg E2tm, ^Ψ^E2tm, or 150 µg ^Ψ^E2tm groups (Fig. 4G). These data suggest that the 150 µg E2tm mRNA vaccine elicited more robust immune responses than 150 µg ^Ψ^E2tm or a lower dose of the E2tm mRNA vaccine.

### E2tm mRNA vaccines protect pigs against the CSFV challenge

**Fig 5:**
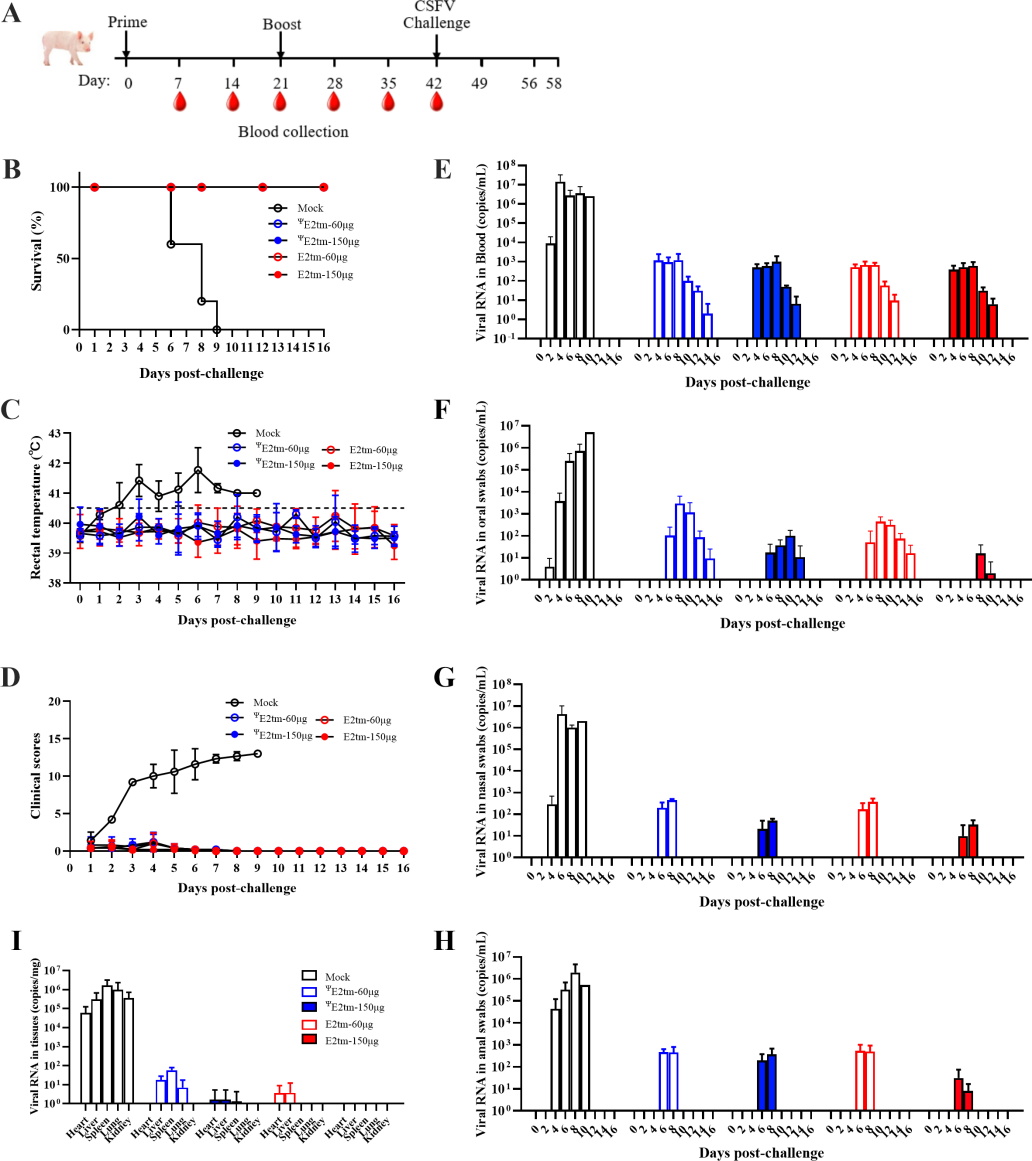
Immunization with E2tm mRNA vaccines provided protection against CSFV infection. (**A**) Schematic diagram of immunization and challenge experiments in pigs. Pigs were immunized with two doses of ^Ψ^E2tm (60 µg and 150 µg) or E2tm (60 µg and 150 µg) mRNA vaccines at 21-day interval. The pigs were challenged with CSFV Shimen strain at 3 weeks post the second immunization. (**B**) Survival rate. (**C**) Rectal temperatures. (**D**) Clinical scores. (**E**) Detection of the viral RNA in the blood samples. (**F**) Detection of the viral RNA in the oral samples. (**G**) Detection of the viral RNA in the nasal samples. (**H**) Detection of the viral RNA in the anal samples. (**I**) Detection of the viral RNA in the tissue samples, including heart, liver, spleen, lung, and kidney. Data were presented as the mean ± SD.

Following the evaluation of the immunogenicity of the E2tm mRNA vaccine, challenge experiments were conducted to determine whether the E2tm and ^Ψ^E2tm mRNA vaccines provide protection against CSFV exposure in pigs. The experimental design is shown in Fig. 5A. All pigs were challenged with 10^6^ TCID_50_ of the CSFV Shimen strain 21 days after the second immunization, and clinical signs and viral load were assessed. At 3 days post-challenge (dpc), the mock-immunized pigs showed elevated temperatures and all pigs died within 9 days (Fig. 5B and C). In contrast, pigs immunized with the E2tm mRNA vaccine showed normal temperatures and all pigs survived the 16-day observation period (Fig. 5B and C). Clinical scores were recorded, as previously described (36). Only one pig’s appetite was affected in the 150 µg E2tm group, while the pigs in the other groups exhibited mild symptoms (Fig. 5D).

The viral RNA shedding profiles of the CSFV-challenged pigs were evaluated. High levels of viral shedding (approximately 10^6^–10^7^ copies/mL) were detected in blood and oral, nasal, and anal swabs of the control group. In contrast, the E2tm mRNA vaccinated group showed significantly reduced viral RNA levels. High-dose mRNA immunization (150 µg ^Ψ^E2tm and E2tm groups) resulted in relatively lower levels of viral RNA detection when compared to the low-dose mRNA immunization (60 µg ^Ψ^E2tm and E2tm groups) (Fig. 5E and F). Approximately 10^3^ copies/mL of viral RNA was detected in the blood of groups 1–4, which decreased gradually, and cleared at 16 dpc in group 1 and 14 dpc in groups 2–4 (Fig. 5E). In the oral swabs, the highest viral shedding was approximately 10^3^ copies/mL in the 60 µg ^Ψ^E2tm group and cleared at 16 dpc in groups 1 and 3, 14 dpc in group 2, and 12 dpc in group 4 (Fig. 5F). In nasal and anal swabs, viral RNA ranging from 10^1^ to 10^3^ copies/mL was detected at 6 and 8 dpc and cleared at 10 dpc in all mRNA-vaccinated groups (Fig. 5G and H). These data suggest that pigs shed significantly less viral RNA in the E2tm mRNA-vaccinated groups than control pigs and viral RNA was quickly cleared, particularly in the 150 µg E2tm group.

At 16 dpc, pigs were euthanized and tissue samples including heart, liver, spleen, lung, and kidney were collected for viral load assessment. Viral RNA was detected in all the tested tissue samples ranging from 10^4^ to 10^6^ copies/mg in the control group (Fig. 5I). Significantly reduced viral RNA levels (10^1^–10^2^ copies/mg) were detected in liver, spleen, and kidney tissue samples in the 60 µg ^Ψ^E2tm group, approximately 10 copies/mg of viral RNA was detected in the heart, liver, and liver tissue samples in the 150 µg ^Ψ^E2tm group. Furthermore, approximately 10 copies/mg of viral RNA was detected in the heart and liver tissue samples in the 60 µg E2tm group. Notably, no viral RNA was detected in any of the tissue samples in the 150 µg E2tm group.

Collectively, these data demonstrated that E2tm mRNA vaccines provided effective protection against CSFV infection in pigs, alleviated clinical symptoms, and significantly decreased viral load. Particularly, viral RNA was completely cleared in pigs immunized with the 150 µg E2tm mRNA vaccine.

## DISCUSSION

Live attenuated vaccines are widely used for CSF control, offering reliable protection due to their high efficacy and low cost. However, these conventional vaccines cannot distinguish between vaccine-induced immunity and natural infection. This has driven the exploration of alternative vaccine strategies, targeting the E2 glycoprotein, an essential surface antigen of CSFV. Recent advances in vaccine technology have yielded various E2-based candidates, including chimeric adenovirus/alphavirus vectored vaccines (37, 38), yeast/baculovirus E2 subunit vaccines (39–41), the plant-derived E2 glycoprotein (42), and self-assembling E2-based nanoparticle vaccine (43). In this study, we used the mRNA platform to design mRNA vaccines targeting the E2 glycoprotein and investigated their protective efficacy in pigs.

First, based on the E2 glycoprotein amino acid sequence, we designed three mRNA constructs: E2_EX lacks the transmembrane region of E2 glycoprotein; E2tm contains a substituted transmembrane region from the PEDV S2 protein; and E2tm-HA incorporates the transmembrane region from influenza virus HA protein (Fig. 1A and B). Previous studies have shown that the native E2 transmembrane region inhibits protein expression due to its high hydrophobic properties (44). Here, the transmembrane region was replaced by those from S2 or HA, which has been known to be expressed (33, 45). To improve protein expression, a previously reported native E2 signal peptide was also added at the N-terminus of the mRNA sequences (46). Following LNP encapsulation, three mRNA vaccine candidates were successfully prepared and expressed in HEK293T cells (Fig. 1C through E). Our results confirmed that transmembrane regions of S2 and HA proteins can be applied for protein expression of E2, which is mostly likely attributed to the hydrophobicity of their transmembrane regions.

Furthermore, we tested the immunogenicity of these three mRNA vaccines in pigs. The E2tm mRNA vaccine induced the highest levels of CSFV-specific and neutralizing antibodies compared to E2_EX and E2tm-HA mRNA vaccines, E2 subunit, and C-strain vaccines (Fig. 2). Notably, the antibodies induced by the E2tm mRNA vaccine were unaffected by maternal-derived antibodies (Fig. 3). These data suggested that the transmembrane region of S2 protein optimally supports E2 immunogenicity.

The efficacy of mRNA vaccines can be influenced by nucleotide modification, such as pseudouridine (Ψ), which enhances mRNA translation efficiency by suppressing Toll-like receptor (TLR) signaling and protein kinase R (PRK) activation (35, 47). However, there is evidence showing that unmodified codon-optimized mRNA may achieve higher protein expression (48). To evaluate this, we prepared Ψ-modified E2tm (^Ψ^E2tm) mRNA vaccine (Fig. 4A and B) and compared the Ψ-modified (^Ψ^E2tm) and unmodified E2tm mRNA vaccines (Fig. 4).

Given that humoral responses may correlate with protective efficacy (49, 50), we first assessed antibody persistence. Our results showed that both formulations induced robust CSFV-specific and neutralizing antibodies. Notably, the 150 µg E2tm group exhibited long-lasting antibody persistence compared to the other groups (Fig. 4C through E). To further improve vaccine durability, several strategies could be explored, such as mRNA sequence engineering including modulating UTRs, ploy(A) tail length and organization, and the coding sequence optimization. The self-replicating mRNA platform could also be utilized to prolong antibody persistence. We further evaluated cellular immune activation by measuring IFN-γ production. The 150 µg E2tm group exhibited superior T cell responses (Fig. 4G). This enhanced cellular immunity could be attributed to the intrinsic immunostimulatory properties of unmodified RNA, which can activate pattern recognition receptor (PRR) (51). PPR activation triggers downstream immune signaling pathways, favorable for vaccine application. This aligns with prior findings demonstrating significantly higher CD8+T cell induction in mice immunized with unmodified mRNA compared to modified mRNA at early time points (52). Subsequent challenge experiments showed that both E2tm and ^Ψ^E2tm mRNA vaccines provided effective protection against CSFV infection, significantly reducing viral shedding and viremia (Fig. 5A through I). Notably, the 150 µg E2tm dose provided optimal protection, effectively suppressing viremia and preventing viral dissemination to tissues, resulting in undetectable viral RNA in swab samples.

While our findings demonstrated robust immunogenicity and effective protection against viral challenge conferred by E2tm vaccines in growing pigs, several limitations should be acknowledged to guide future investigation, such as safety evaluation in piglets and pregnant sows, determination of the minimum effective dose to establish efficient vaccination efficacy, evaluation of single-dose efficacy, and comparative challenge studies with conventional vaccines (C-strain and E2 subunit vaccines). Furthermore, the mechanisms underlying the enhanced immunogenicity of unmodified E2tm mRNA vaccine require further investigation. Regarding practical implementation, while recent advances in lyophilization technology have improved thermostability for certain mRNA vaccines (53–55), the current cold chain requirements remain significant challenges for veterinary applications. These factors represent important practical barriers that require further research and development.

In summary, we successfully developed three mRNA vaccines expressing the CSFV E2 glycoprotein, of which the E2tm mRNA vaccine demonstrated superior immunogenicity. Further investigation revealed that both Ψ-modified and unmodified E2tm mRNA vaccines induced durable antibody responses and cellular immunity, providing effective protection against the CSFV challenge. Particularly, the 150 µg E2tm dose exhibited optimal immune responses and protection efficacy. Thus, we present a CSFV mRNA vaccine candidate that could be further optimized and used as an alternative CSFV vaccine to prevent CSFV infection.

## MATERIALS AND METHODS

### Cell line and virus strains

HEK293T cells and PK15 cells were grown in Dulbecco’s modified Eagle’s medium (Gibco) supplemented with 10% fetal bovine serum (FBS) (Gibco), 100 U/mL penicillin, 100 µg/mL streptomycin, and maintained at 37°C with 5% CO_2_. The CSFV Shimen, HuB100, and HeBHH1/95 strains were used in this study.

### Design of E2 plasmids

The E2 glycoprotein of the CSFV Shimen strain (GenBank accession no. AF092448.2) was used as the reference amino acid sequence. Three mRNA sequences were designed as follows: E2_EX, E2tm, and E2tm-HA. A previously reported native signal peptide of E2 (MKVLRGQIVQGVIWLLLVTGAQG) was added to the N terminus of the mRNA sequences (46). Specifically, E2_EX comprised a signal peptide and the ectodomain of E2 glycoprotein. E2tm incorporated a signal peptide, the ectodomain of the E2 glycoprotein, and transmembrane region of the PEDV S2 protein. E2tm-HA contained a signal peptide, the ectodomain of the E2 glycoprotein, and transmembrane region of the influenza virus HA protein. These sequences were cloned into the plasmid with backbone elements (T7 promoter, 5′ and 3′ UTR, poly(A) tail), as described previously (56).

### Generation of E2 mRNAs and mRNA-LNP

Linearized plasmids were used to generate mRNAs. Following plasmid linearization, the mRNAs were synthesized via *in vitro* transcription using T7 RNA polymerase, and cap1 analog was enzymatically added to the mRNAs (APExBIO). For E2tm mRNA, nucleotides with a global substitution of uridine with pseudouridine (Ψ, APExBIO) were used to generate the modified ^Ψ^E2tm mRNA. The mRNAs were purified through two chromatographic procedures and further processed for RNA integrity and quality control analysis (56). Subsequently, the mRNAs were encapsulated into LNP, as described previously (56). All the LNP encapsulated mRNAs were tested for particle size and polymer dispersity index (PDI).

### Expression of mRNA encoding E2 glycoprotein

The expression of E2 mRNA-LNPs was evaluated in HEK293T cells. HEK293T cells were seeded in 6-well plates. The next day, the HEK293T cells were incubated with 2 µg of E2_EX, E2tm, or E2tm-HA mRNA-LNPs. After 24 h incubation, protein expression was analyzed using western blotting and immunofluorescence assay (IFA).

### Animal immunization and viral challenge

To assess the antibodies induced by the E2tm, E2_EX, and E2tm-HA mRNA vaccines, twenty 30-day-old, CSFV-negative pigs were randomly divided into five groups (*n* = 4) and immunized intramuscularly with 60 µg (in 1 mL PBS) of E2_EX, E2tm, E2tm-HA mRNA vaccines, or a commercial E2 subunit vaccine from Jinyu Group, or 10^4.5^ TCID_50_ the live attenuated C-strain vaccine. A booster immunization was conducted 21 days after primary immunization for the mRNA and E2 subunit vaccines. Sera was collected at 7, 14, 21, 28, and 35 dpi for antibody analysis.

To investigate the influence of maternal antibodies on CSFV-specific antibody responses to E2tm mRNA, sixteen 7-day-old piglets were randomly divided into four groups (*n* = 4): E2tm mRNA vaccine, C-strain, E2 subunit vaccine, and mock groups. Pigs were immunized intramuscularly with 60 µg (in 1 mL PBS) E2tm mRNA or E2 subunit vaccines or C-strain. Pigs from the mock group were intramuscularly injected with 1 mL PBS. Sera was collected at 0, 7, 14, and 21 dpi for antibody analysis.

To assess the impact of nucleotide modification on E2tm mRNA, forty-one 30-day-old, CSFV-naive pigs were randomly divided into five groups. Groups 1 and 2 (*n* = 9) were immunized intramuscularly with 60 and 150 µg (in 1 mL PBS) of ^Ψ^E2tm mRNA vaccine, respectively. Groups 3 and 4 (*n* = 9) were immunized intramuscularly with 60 and 150 µg (in 1 mL PBS) of E2tm mRNA vaccines, respectively. Group 5 (*n* = 5) was used as a control and was intramuscularly injected with 1 mL PBS. A booster immunization was conducted 21 days after primary immunization. Serum samples were collected from pigs in groups 1–4 at 0, 7, 14, 21, 28, 35, 42, 63, 84, and 108 dpi for antibody detection. At 28 dpi, serum samples were also collected for the isolation of PBMCs for ELISpot assay and assessment of neutralizing activity against different CSFV strains. At 42 dpi, five pigs from groups 1–4 were randomly selected for viral challenge. Five pigs in the mock group served as controls. Pigs were challenged with 1 mL of the CSFV Shimen strain (10^6^ TCID_50_/mL) and monitored daily for rectal temperatures and clinical signs. Clinical samples of serum and oral, nasal, and anal swabs were collected at 0, 2, 4, 6, 8, 10, 12, 14, and 16 dpc. All surviving pigs were euthanized and necropsied 16 dpc. Tissue samples from the heart, liver, spleen, lungs, and kidneys were collected to assess viral load.

### Enzyme-linked immunosorbent assay

CSFV-specific antibodies were evaluated using a blocking ELISA Kit according to the manufacturer’s instructions (IDEXX). Briefly, the pre-coated plates with purified CSFV E2 glycoprotein were incubated with serum samples for 2 h at RT. After washing, HRP-conjugated anti-pig antibody was added to the plates for 30 min at RT. The plates were then incubated with the TMB substrate for 10 min at RT. Reactions were terminated using 2 M sulfuric acid, and absorbance was recorded at 450 nm. The results were considered positive when the S/P ratio was ≥40%.

### Serum neutralization assays

The collected serum samples were heat-inactivated for 30 min at 56°C. Subsequently, the serum samples were serially diluted in twofold increments. Next, 100 µL of the diluted samples was mixed with an equal volume of CSFV Shimen or HuB100 or HeBHH1/95 strain (200 TCID_50_) and incubated for 1 h at 37°C. The mixtures were added to the PK15 cells cultured in 96-well plates. After 72 h incubation, the cells were washed three times with PBS and then fixed with ice-cold acetone: methanol (1:1, vol) for 1 h. After fixation, cells were incubated with E2 monoclonal antibody prepared by China Institute of Veterinary Drug Control, followed by incubation FITC labeled anti-mouse antibody (Sigma). The results were observed under a fluorescence microscope. The virus neutralizing antibody titers were presented as the highest serum dilution that protected >50% of the cells from infection (ND_50_). Serum was considered neutralizing antibody-positive when the titer was ≥10 ND_50_.

### ELISpot assay

Porcine INF-γ ELISpot assay was conducted on PBMCs, following the manufacturer’s instructions (MabTech). PBMCs were resuspended in RPMI 1640 medium supplemented with 10% FBS, 100 U/mL penicillin, and 100 µg/mL streptomycin. ELISpot plates were blocked and then stimulated with E2 glycoprotein (2 µg), ConA (4 µg), and PBS. Then, 5 × 10^5^ PBMCs were added to each well and incubated overnight. After three washes with PBS, the plates were incubated with the detection antibody followed by incubation with streptavidin-ALP. The BCIP/NTB-plus substrate was then added until distinct spots emerged. Spots indicating antigen-specific IFN-γ secreting cells were recorded.

### Quantification of CSFV RNA by quantitative real-time PCR

CSFV RNA loads in samples from blood; oral, nasal, and anal swabs; and tissue samples, including the heart, liver, spleen, lungs, and kidneys were determined by quantitative real-time PCR (RT-qPCR) using HiScript III All-in-one RT SuperMix Perfect (Vazyme). The qPCRs were performed using the forward primer: 5′-TACAGGACAGTCGTCAGT-3′, reverse primer: 5′-CCGCTAGGGTTAAGGTGTGTCT-3′, and probe: 5′-FAM-CCCACCTCGAGATGCTATGTGGACGA-TAMRA-3′. The reactions were conducted using an ABI QuantStudio v.5 Pro thermocycler.

### Western blotting

HEK-293T cells cultured in 6-well plates were incubated with the mRNA-LNP vaccines for 24 h. After 24 h incubation, HEK293T cells treated with E2 mRNA-LNPs were harvested and lysed. Equal amounts of HEK293T cell lysates were separated under reducing conditions on sodium dodecyl sulfate-polyacrylamide gel electrophoresis (SDS-PAGE) gels. Proteins were transferred onto polyvinylidene difluoride membranes. Membranes were blocked with 3% bovine serum albumin (BSA) in Tris-buffered saline buffer containing 1% Tween 20 (TBST) and then incubated with a mouse anti-E2 monoclonal antibody (prepared by China Institute of Veterinary Drug Control). Membranes were washed with TBST and incubated with anti-mouse horseradish peroxidase (HRP)-conjugated antibody (Invitrogen). Finally, the membranes were developed using a chemiluminescent substrate (Thermo Fisher Scientific). Membranes were detected using a chemiluminescence image analysis system (Tanon 5200). GAPDH was used as a loading control.

### Immunofluorescence assay

HEK293T cells treated with E2 mRNA-LNPs were washed with PBS and fixed with 4% paraformaldehyde for 15 min at room temperature (RT). After fixation, cells were washed with PBS and blocked in 3% BSA in PBS for 2 h at RT. Cells were incubated with mouse anti-E2 antibody prepared by China Institute of Veterinary Drug Control. Then, the cells were washed with PBS and incubated with Alexa Fluor-488-conjugated anti-mouse antibody (Sigma). Finally, cells were examined under a fluorescence microscope (Nikon).

### Statistical analysis

GraphPad Prism 8.0 was used for graphical and statistical analysis. Data are presented as the mean ± standard deviation (SD). The results from different groups were analyzed using one-way or two-way ANOVA with Turkey’s multiple comparison test. Statistical significance was set at *P* < 0.05 (ns, not significant, *P < 0.05*,* **P < 0.01, ***P < 0.001, ****P < 0.0001).

## Data Availability

All data associated with this study are available within the paper and from the corresponding authors upon reasonable request.
